# An evaluation study of biclusters visualization techniques of gene expression data

**DOI:** 10.1515/jib-2021-0019

**Published:** 2021-10-27

**Authors:** Haithem Aouabed, Mourad Elloumi, Rodrigo Santamaría

**Affiliations:** Laboratory of Technologies of Information and Communication, and Electrical Engineering (LaTICE), University of Tunis, Tunis, Tunisia; Faculty of Economic Sciences and Management of Sfax, University of Sfax, Sfax, Tunisia; Faculty of Computing and Information Technology, The University of Bisha, Bisha, Saudi Arabia; Departamento de Informática y Automática, Universidad de Salamanca, Salamanca, Spain

**Keywords:** biclustering algorithms, biclusters, information visualization, overlaps, visualization

## Abstract

*Biclustering* is a non-supervised data mining technique used to analyze gene expression data, it consists to classify subgroups of genes that have similar behavior under subgroups of conditions. The classified genes can have independent behavior under other subgroups of conditions. Discovering such co-expressed genes, called *biclusters*, can be helpful to find specific biological features such as gene interactions under different circumstances. Compared to clustering, biclustering has two main characteristics: *bi-dimensionality* which means grouping both genes and conditions simultaneously and *overlapping* which means allowing genes to be in more than one bicluster at the same time. Biclustering algorithms, which continue to be developed at a constant pace, give as output a large number of overlapping biclusters. Visualizing groups of biclusters is still a non-trivial task due to their overlapping. In this paper, we present the most interesting techniques to visualize groups of biclusters and evaluate them.

## 1 Introduction

*Gene expression data* are the product of microarrays. Tens of thousands of genes are represented by a matrix where a row represents a gene, a column represents a condition and a cell represents the expression level of the corresponding gene under the corresponding condition. *Clustering* consists of selecting genes (rows) with similar expression patterns under the whole set of conditions (columns) [1]. Many clustering techniques, such as *hierarchical clustering* [2] and *k-means* clustering [3], have been successfully applied in the analysis of gene expression data. However, the detection of new insights from biological data in specific situations, such as finding genes involved in tumor progression, identifying functions of new genes or identifying new therapeutic strategies, needs to carry out the clustering on both dimensions simultaneously, i.e., genes and conditions. Indeed, several genomic data analysis workflows involve the identification of groups of biological entities, e.g., genes that exhibit similar behavior under certain conditions. These nuggets of biological knowledge can be discovered thanks to a machine learning technique, called *biclustering*. It was adopted for the first time by Cheng and Church [4] to analyze expression data. Compared to clustering, biclustering has two main features: *bi-dimensionality*, which means grouping both genes and conditions simultaneously, and *overlapping*, which means allowing genes to be in more than one bicluster at the same time. These features illustrate how genes can take part to more than one activity. Biclustering algorithms continue to be developed at a constant pace with the aim to find multiple biclusters which are generally with high rates of overlapping. Valente-Freitas et al. [5] made an interesting survey on different biclustering algorithms.

Visualizing groups of biclusters is a very good way to infer patterns from gene expression data [6]. However, given the special features of biclustering, i.e., bi-dimensionality and overlaps, its application to gene expression data often generates a wide number of overlapping groups of biclusters, which are very hard to represent in an informative way in a single eyesight. Indeed, representing groups of biclusters in a clear visual representation is not a trivial task. Discovering new insights from large and complex multi-dimensional datasets needs a good combination of data processing algorithms and interactive visualization techniques [7–11]. This combination is successfully applied on groups of biclusters of gene expression data [12]. The most popular techniques to visualize a single bicluster are *heatmaps* [1] and *parallel coordinates* [13]. The difficulty arises when we want to visualize more than one bicluster on the same screen, at the same time.

In this paper, we present the most interesting techniques to visualize groups of biclusters and evaluate them. The rest of this paper is organized as follows: in Sections 2 and 3, we present some preliminaries, respectively, on biclustering of gene expression data and information visualization. In Section 4, we present biclustering visualization techniques. In Section 5, we make an evaluation of biclustering visualization techniques. Finally, in the last section we present our conclusion.

## 2 Biclustering of gene expression data

Let’s start by some definitions.

### 2.1 Definitions

A *bicluster* is a subset of genes that behave similarly under a subset of conditions. Behaving similarly means that all the genes in the bicluster have expression levels within the same range, or that the expression varies in the same fashion along the conditions [14]. Note that biclusters can *overlap* which means that one or more genes and/or conditions can belong to more than one bicluster.

Formally, a bicluster can be defined as follows: Let *I* = {1, 2, …, *n*} be a set of indices of *n* genes, *J* = {1, 2, …, *m*} be a set of indices of *m* conditions and *M*(*I*,*J*) be a data matrix associated with *I* and *J*. A bicluster associated with the data matrix *M*(*I*,*J*) is a couple (*I*′,*J*′) such that *I*′ ⊆ *I* and *J*′ ⊆ *J*.

The *biclustering problem* can be formulated as follows: Given a data matrix *M*, construct a group of biclusters *B*_opt_ associated with *M* such that:

(1.1) $$f\left(B_{\text{opt}}\right)=\underset{B\in BC\left(M\right)}{\max}f\left(B\right)$$

where *f* is an objective function measuring the *quality*, i.e., degree of coherence, of a group of biclusters and *BC*(*M*) is the set of all the possible groups of biclusters associated with *M* [15, 16]. Biclustering is an NP-hard problem [4, 17].

### 2.2 Types of biclusters

**Figure 1:**
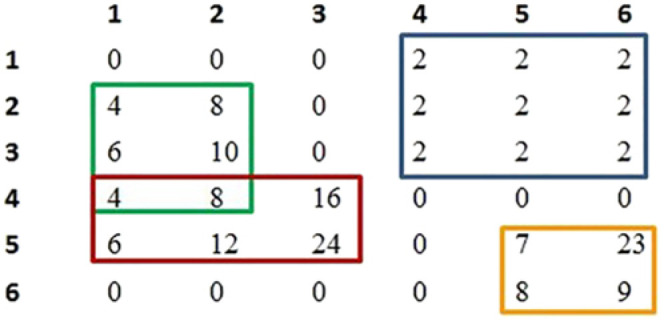
Four biclusters from a simplified expression matrix. The blue bicluster is a bicluster with *constant values*. The green one is a bicluster with *additive coherent values*. The red one is a bicluster with *multiplicative coherent values*. And the orange one is a bicluster with *coherent evolution* by columns only.

**Figure 2:**
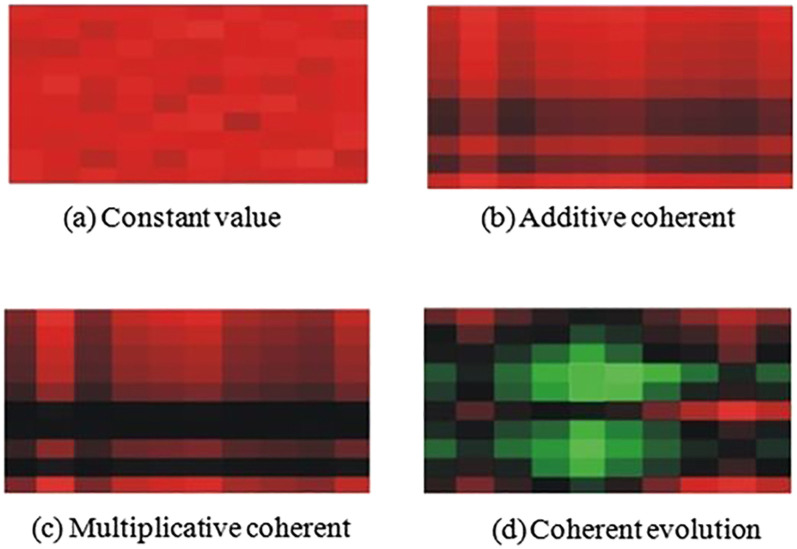
Different types of biclusters shown as heatmaps, where the red color encodes high expression levels and the green one encodes low expression levels [14].

A bicluster can be in one of the following main classes [17] (see Figures 1 and 2):

– Bicluster with *constant values*: It is a bicluster where all the values are equal to a constant *c*:
(1.2) $$m_{ij}=c$$
– Bicluster with *constant or coherent values on rows*: It is a bicluster where all the expression levels can be obtained by using one of the following equations:
(1.3) $$m_{ij}=c+a_{i}$$
(1.4) $$m_{ij}=c*a_{i}$$
  where *c* is a constant and *a*_*i*_ is the adjustment for the row *i*, 1 ≤ *i* ≤ *n*.
– Bicluster with *constant or coherent values on columns*: It is a bicluster where all the expression levels can be obtained by using one of the following equations:
(1.5) $$m_{ij}=c+b_{j}$$
(1.6) $$m_{ij}=c*b_{j}$$
  where *c* is a constant and *b*_*j*_ is the adjustment for the column *j*, 1 ≤ *j* ≤ *m*.
– Bicluster with *coherent values*: There are two types of biclusters with *coherent values*. Those with *additive model* and those with *multiplicative model* defined respectively by:
(1.7) $$m_{ij}=c+a_{i}+b_{j}$$
(1.8) $$m_{ij}=c*a_{i}*b_{j}$$
  Some authors call these factors *shifting* and *scaling factors*, respectively [18].
– Bicluster with *coherent evolution*: It is a bicluster where all the rows (resp. columns) induce a linear order across a subset of columns (resp. rows).

### 2.3 Groups of biclusters

**Figure 3:**
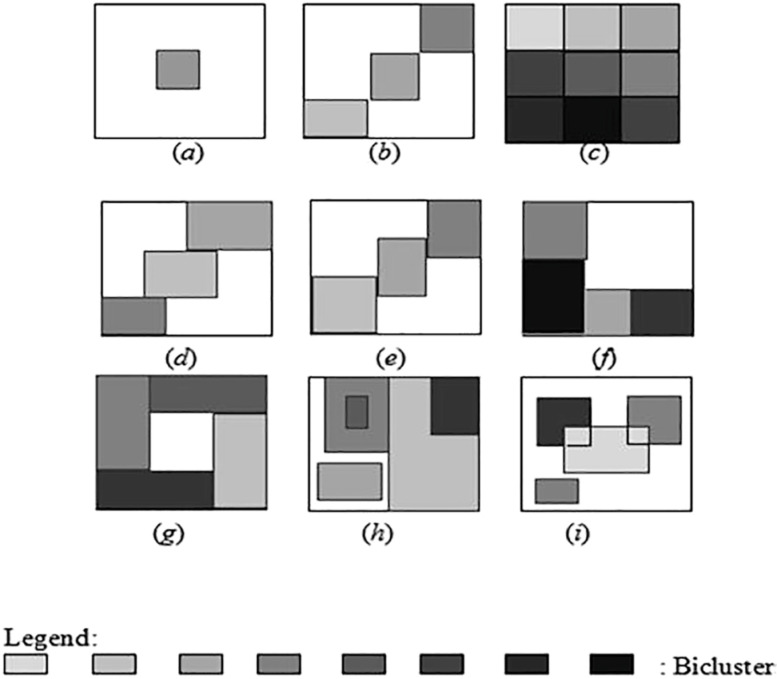
Types of groups of biclusters [15].

A group of biclusters can be one of the following types [17]:

– *Single bicluster* (Figure 3(a)).
– *Exclusive rows and columns group of biclusters* (Figure 3(b)).
– *Non-overlapping group of biclusters with checkerboard structure* (Figure 3(c)).
– *Exclusive rows group of biclusters* (Figure 3(d)).
– *Exclusive columns group of biclusters* (Figure 3(e)).
– *Non-overlapping group of biclusters with tree structure* (Figure 3(f)).
– *Non-overlapping non-exclusive group of biclusters* (Figure 3(g)).
– *Overlapping group of biclusters with hierarchical structure* (Figure 3(h)).
– *Arbitrarily positioned overlapping group of biclusters* (Figure 3(i)).

## 3 Information visualization

*Information Visualization* (InfoVis) is a research field based on interactivity to explore insights from data by the use of visualization methods and techniques. In the literature, there is more than one definition of InfoVis:

– It is the use of computer-supported, interactive visual representations of data to amplify cognition [19].
– Another definition, InfoVis is defined as the science of visual representation of data, defined as information which has been abstracted in some schematic form, including attributes or variables for the units of information [20].
– InfoVis is also defined as a method for representing data accurately on the web and elsewhere. It gives a unique perspective on the data set. It is a representation of data in a visual context, which helps to understand the significance of data [21].
– An interesting definition of InfoVis is presented in [22] where InfoVis is a cognitive process used to analyze and represent data, to have a better understanding of a situation and have an opportunity to act upon that understanding. It also enables effective communications and presentations, and hence have a gain in insights, not simply to view pictures.

In order to design a good visualization method, analysts need to know the data type to be visualized [23] which can be one of the following types [14]:

– *1-dimensional*: data organized in a sequential order, such as biological sequences.
– *2-dimensional*: planar data, such as expression matrices.
– *3-dimensional*: real-world objects, such as biological macromolecules.
– *n-dimensional*: data with *n* > 3 variables, such as relational databases.
– *Temporal*: data with start and end times, such as processes or events.
– *Trees*: data where each item is linked to one parent, such as phylogenetic trees.
– *Networks*: data where each item is linked to an arbitrary number of other items, such as gene regulation networks.

## 4 Biclustering visualization techniques

Let’s start by heatmaps.

### 4.1 Heatmaps

**Figure 4:**
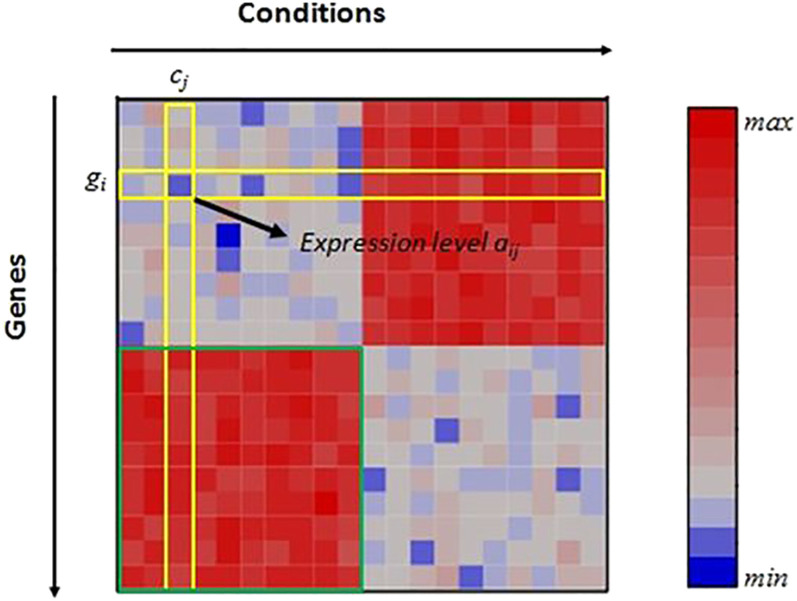
Heatmap representation of gene expression data. In green, a captured bicluster. In yellow, expression level *a*_*ij*_ corresponding to a gene *g*_*i*_ and a condition *c*_*j*_.

A *heatmap* is a two-dimensional visualization technique that displays values of data in a matrix. In the case of gene expression data, the *x*-axis represents the conditions (columns) and the *y*-axis represents the genes (rows). A cell *a*_*ij*_ that represents the expression level of the *i*th gene under the *j*th column is drawn as a small square (pixel) colored based on a defined color scale. Often green, red and black colors are used in order to match with the typical fluorescent dyes in DNA microarrays. Often, green color indicates low expression level, red color indicates high expression level, and black color indicates unchanged expression level. In order to draw a bicluster, its corresponding rows and columns are rearranged and usually placed in the upper left corner [24]. Techniques like *reordering* or *replication* are used to visualize more than one bicluster in a global view. Figure 4 shows an example of a heatmap.

#### 4.1.1 Reordering

**Figure 5:**
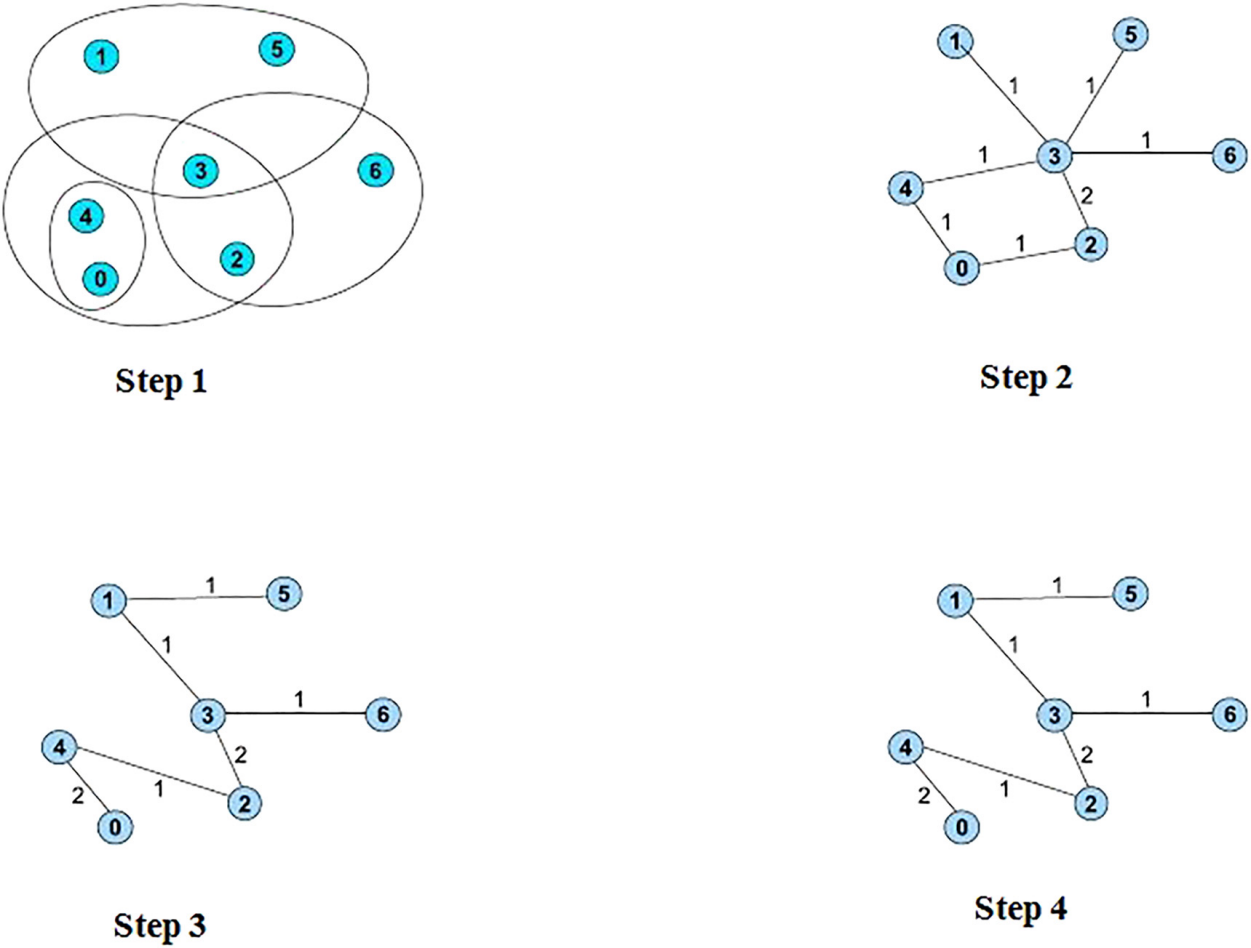
A running example of the reordering algorithm [25].

In order to draw several biclusters at the same view, reordering can be a solution for heatmaps representation. Several algorithms were developed in the literature. Jin et al. [25] defined a heuristic iterative algorithm that formulated the overlapping biclusters visualization problem as an optimization problem. The algorithm defines a reordering approach that exploits analogies to the *hypergraph vertex ordering* problem which is a generalization of the traditional *minimal linear arrangement* or *graph ordering* problem. First, the heatmap matrix is converted to a *hypergraph* that is transformed to a weighted undirected graph based on a start order in one of three defined structures: a *path*, a *cycle* or a *multi-cycle*. Second, the *Minimum Linear Arrangement* problem algorithm (MinLA) is applied to the constructed graph to find a good new order. Third, the *hypergraph* is converted again to another graph based on the new order. The process is iterated until a good enough order is found or there are no more possible improvements. Figure 5 illustrates a running example of the described algorithm.

**Figure 6:**
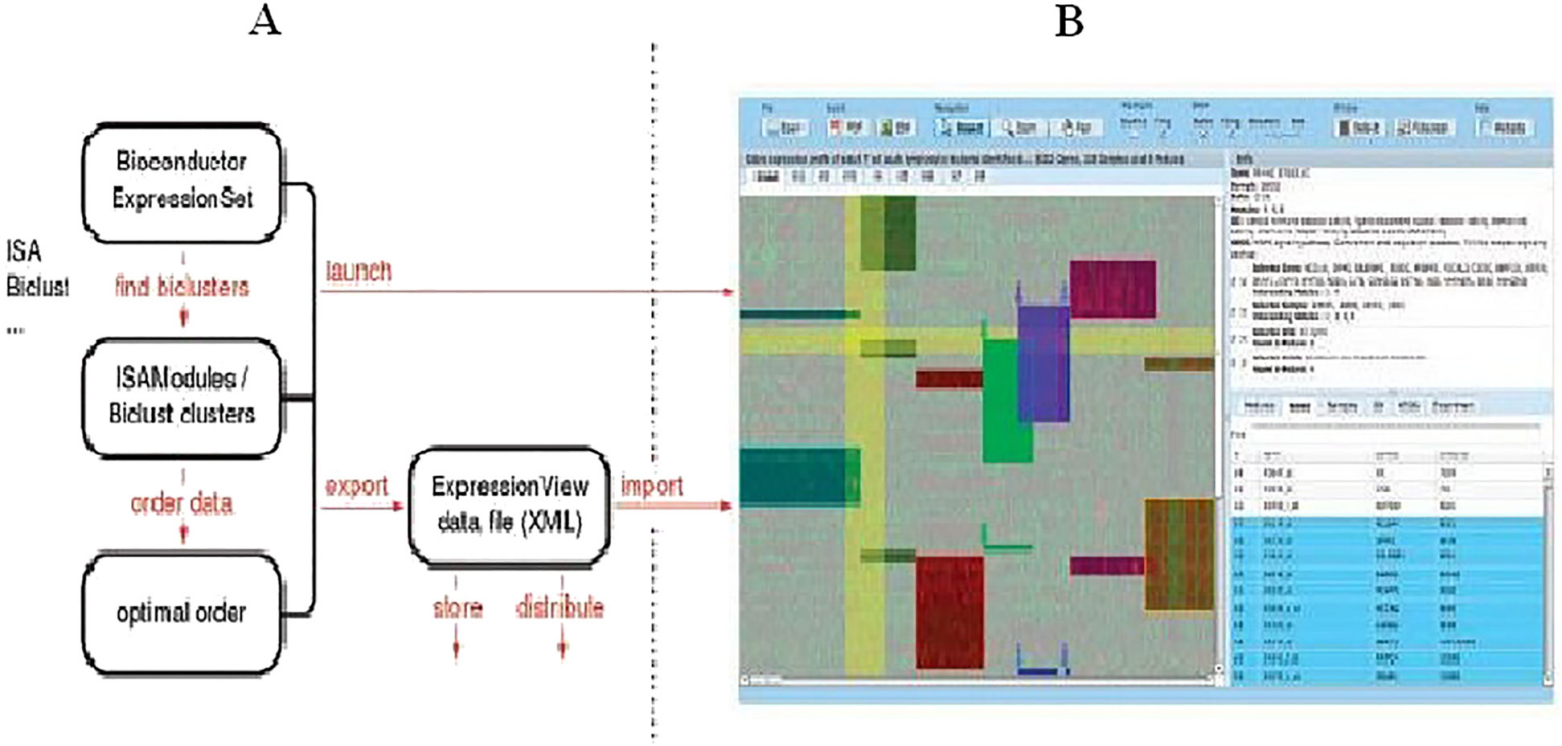
Workflow of Luscher et al. [26] algorithm. (A) Analysis part in which the reordering algorithm is executed with other analysis processes. (B) Heatmap visualization of the biclustering results.

In Luscher et al. [26], they propose an algorithm that consists of defining an optimal arrangement way that maximizes the areas of the largest contiguous parts of biclusters obtained from a gene expression matrix. As a start point, the data is represented as a binary matrix where the number of rows corresponds to the number of genes or conditions and the number of columns corresponds to the number of biclusters. The reordering strategy is applied independently on rows and columns in order to maximize as much as possible the quality of drawn biclusters. The optimizing order is done in four steps: the first step, called *simplify*, excludes duplicated rows to alleviate the complexity of the problem. The second step, called *prearrange*, consists of finding a good starting point for the optimization by creating a new order, starting from the first row and adding the consecutive rows one after the other, placing them at the optimal position. The third step, called *arrange*, is the main step of the algorithms. It consists of maximizing an alignment score based on a greedy approach that, on one side, moves parts of a given bicluster to better positions and, on the other side, permutes elements (genes or conditions) contained in a given bicluster. The last step, called *complexity*, restores the original dimensions of the problem by inserting the removed rows in their new positions. Figure 6 shows a workflow of the proposed technique.

#### 4.1.2 Duplication

For some special cases in heatmaps representation, showing all biclusters based on reordering techniques is not enough. Indeed, rows and columns need to be duplicated in order to show biclusters as contiguous blocks in the same heatmap. In the literature, some propositions applied this strategy.

**Figure 7:**
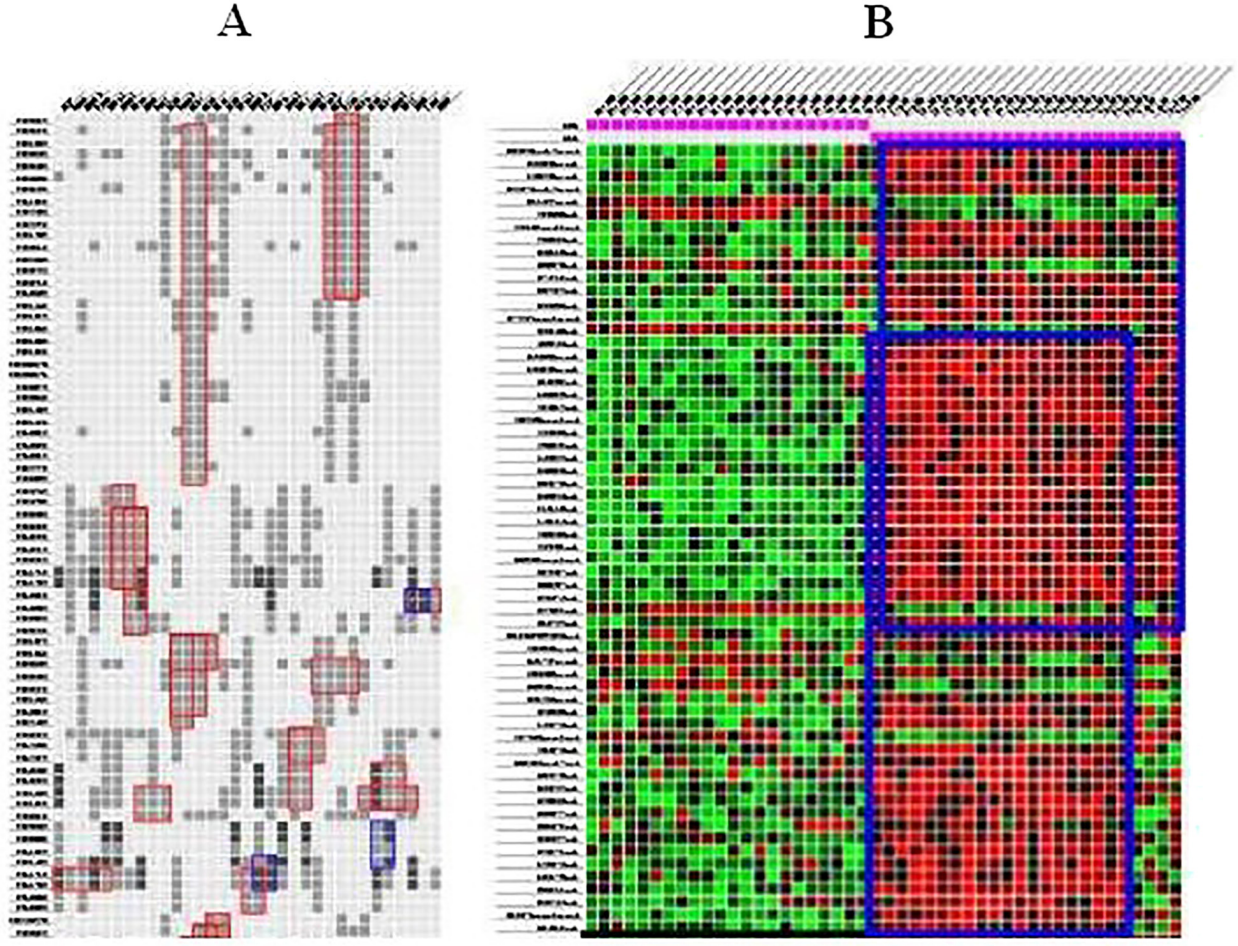
Several biclusters visualized based on Grothaus et al. [27] technique. (A) Red rectangles. (B) Blue rectangles.

Grothaus et al. [27] proposed an algorithm to visualize biclusters and their possible overlaps as contiguous areas in the same heatmap. The main principle of their algorithm is repeating rows and columns to be able to draw overlapping biclusters. The proposed strategy is inspired from the *hypergraph superstring problem* [28]. The algorithm defines a method to minimize the number of duplicated rows and columns. It is applied to rows and columns independently. It uses a data structure, called *PQ tree* [29], which is used to define all possible columns to be consecutive with duplication, if that is necessary, in order to construct contiguous biclusters and a list of *REDUCE* operations, which are used to hierarchically cluster the rows in order to improve the quality of the final results. An example with two different expression matrices is illustrated in Figure 7.

**Figure 8:**
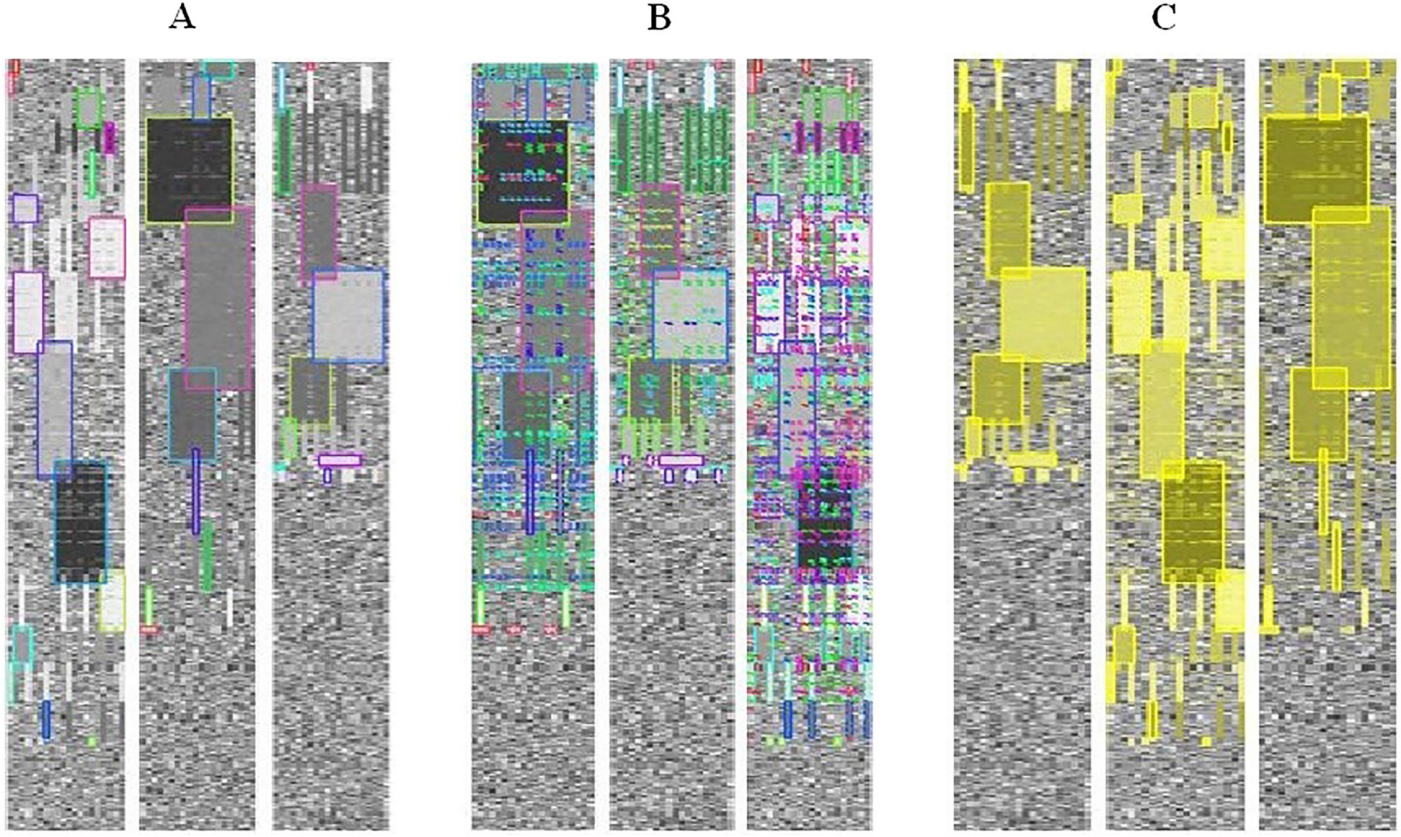
Biclustering visualization for three expression datasets. (A) Each bicluster is represented by its main rectangle. (B) All biclusters are represented. (C) Representation with highlighted biclusters [30].

In the work of Heinrich et al. [30], the authors defined a biclustering layout algorithm and an interactive visualization tool to represent multiple biclusters. The first step in their algorithm consists of mapping the heatmap to grayscale values using *linear interpolation* between the smallest and the largest value of the data matrix. Then, a distinct color is assigned to each bicluster. In the case of selection, biclusters are colored with a transparent yellow color which is blended additively in overlapping regions but the user always has the possibility to choose his own colors. In order to allow analysts to interactively decide which biclusters to visualize contiguously, the algorithm uses the reordering as well as replication techniques of rows and/or columns. This interactivity of this method minimizes, with a high rate, the number of duplicates and increases a little bit the scalability of the method. Figure 8 shows the different representations of heatmaps for three expression datasets.

Despite the fact that heatmaps visualization is the most widespread technique that is used to represent single biclusters, it suffers from a geometrical limitation, especially, when used to display biclusters with high rates of overlaps.

### 4.2 Parallel coordinates

**Figure 9:**
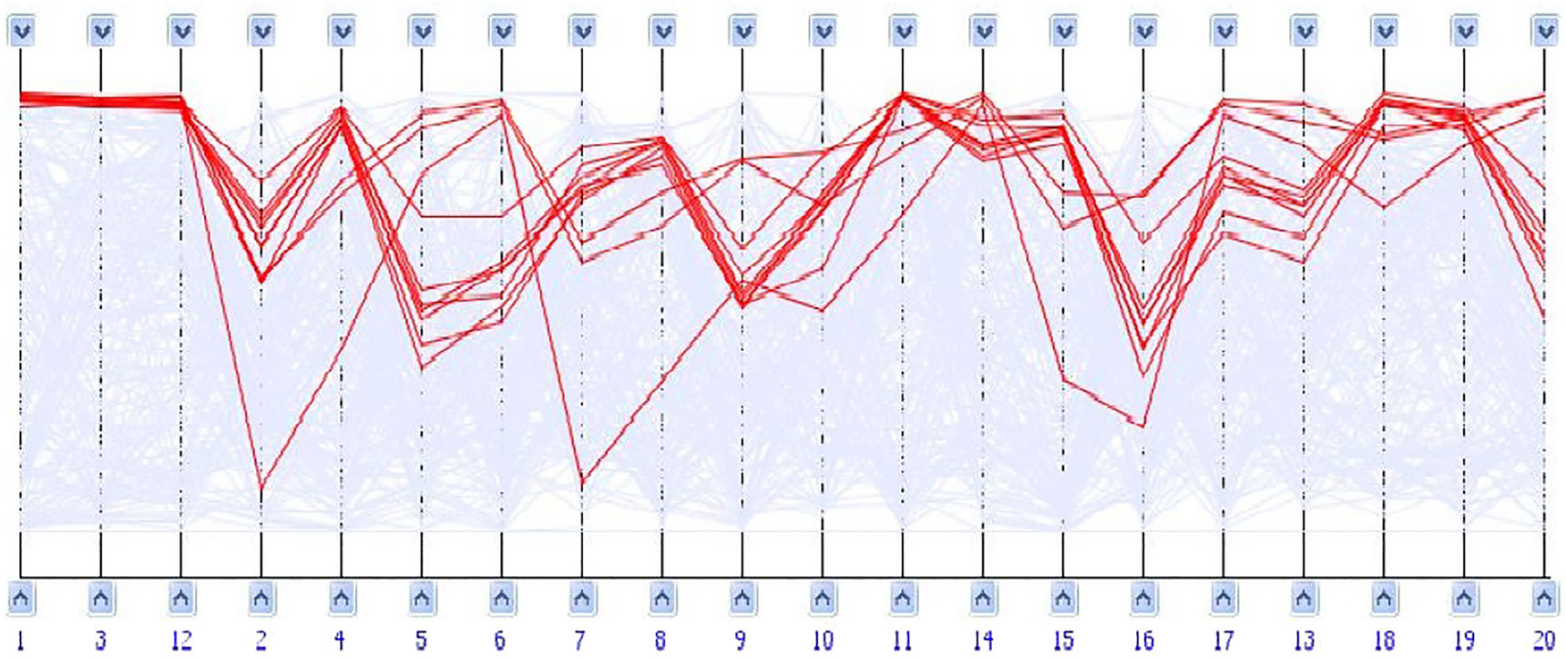
Parallel coordinates visualization. Polylines of interesting genes colored in red [31].

*Parallel coordinates* technique is a visualization technique used to plot high-dimensional multivariate data. Each dimension corresponds to a *vertical axis* and each data element is posted as a series of connected points forming a *polyline* along the defined axes. This technique has been also used to visualize gene expression data. To show gene profiles in an *m-dimensional* space, a backdrop is drawn consisting of *m* parallel lines, vertical and equally spaced, that represent different conditions. Each gene profile is represented as a polyline of *m* points displayed on the parallel axes. The position of a point matches its corresponding expression level. Figure 9 illustrates an example of parallel coordinates.

**Figure 10:**
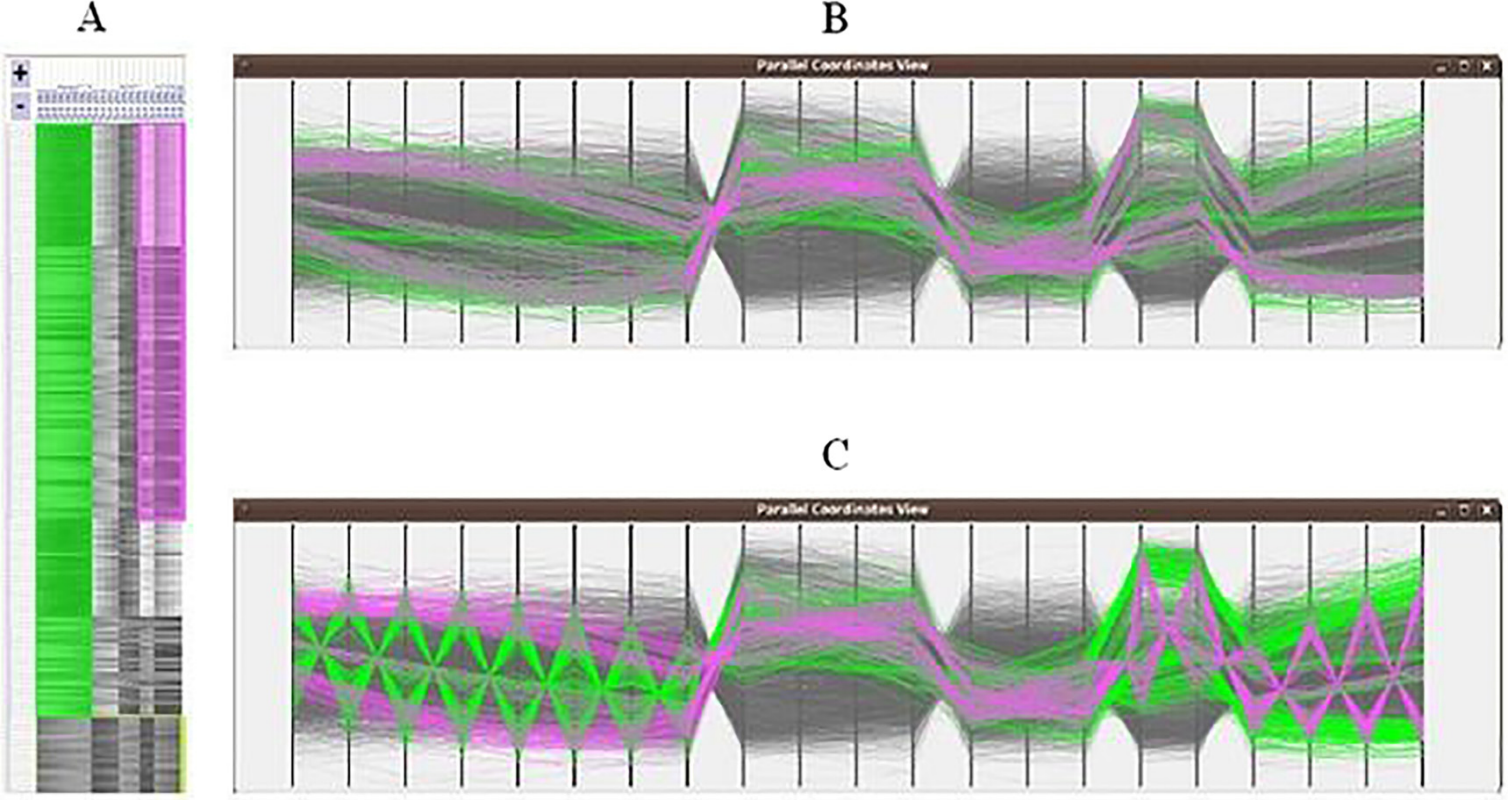
Two biclusters visualized as a heatmap then mapped to parallel coordinates. (A) Heatmap of the two biclusters. (B) The same two biclusters visualized as parallel coordinates without centroids. (C) The same two biclusters visualized as parallel coordinates with centroids [30].

In order to visualize more than one bicluster in the same view using parallel coordinates, Heinrich et al. [30] tried to do several transformations on their heatmap representation in order to be compatible with parallel coordinates features. The rows of the matrix become the lines in the parallel coordinates plot. The horizontal axes are arranged in the same order as the columns in the heatmap representation. In order to visualize the conditions of genes belonging to a bicluster, the proposed technique calculates the average vertical position of all lines of a bicluster and defines points called *centroids*. Then, the lines of the corresponding bicluster are forced to cross these points. As a next step, all biclusters are drawn and colored in a transparent black color. The same colors, used to color biclusters in the heatmap representation, are used in the parallel coordinates plot. To alleviate the cluttering problem, black lines that do not belong to the selected biclusters can be faded out by the user. Figure 10 shows an example of parallel coordinates plot for two biclusters.

Parallel coordinates technique is a good choice to draw large and/or single biclusters. However, cluttering of polylines caused by overlaps between biclusters decreases the efficiency of this technique, to show several biclusters in the same view.

Usually, scalability is the principal drawback of heatmaps and parallel coordinates either because of the large number of biclusters, or because of high overlap rates [31].

### 4.3 Bubble maps

**Figure 11:**
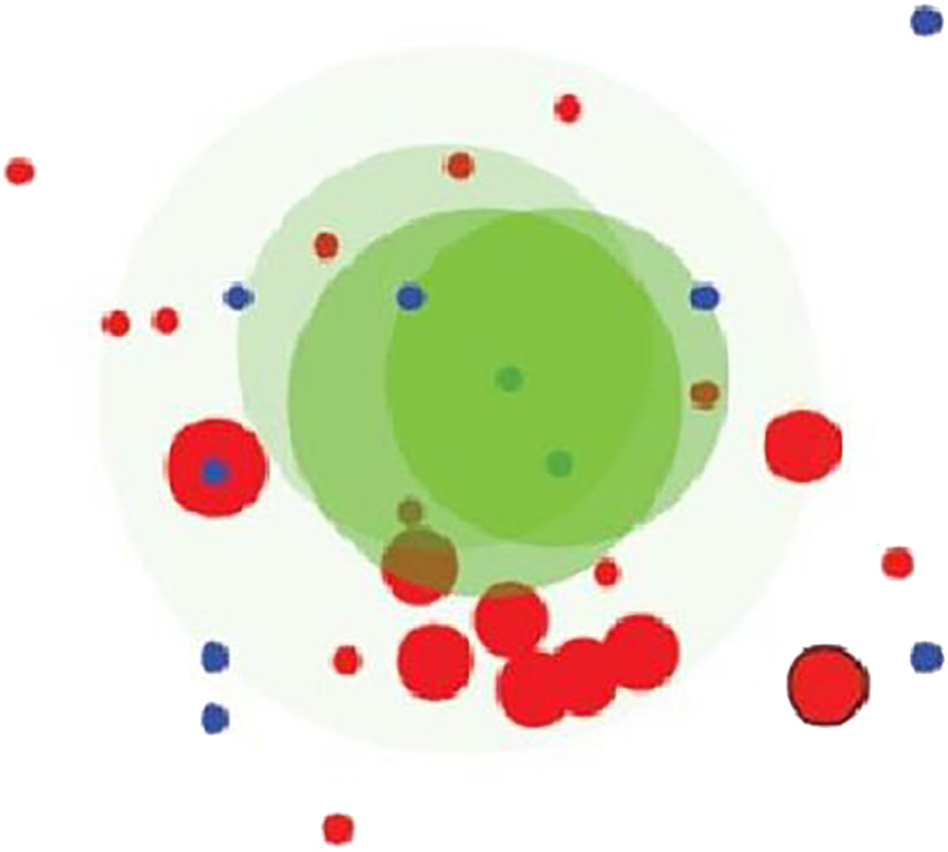
Bubblemap representation of the results of three biclustering algorithms [14].

This technique was proposed by Santamaría et al. [31] and Kaiser et al. [32], it consists of drawing biclusters as circles (bubbles). A color represents a group of biclusters generated by a biclustering algorithm. The algorithm can show up to three groups of biclusters simultaneously. Brightness reflects the bicluster homogeneity. Size represents the size of the bicluster, it is calculated by multiplying the number of genes by the number of conditions. The position of a bubble depends on a 2D projection of two multidimensional points, formed by the rows and the columns, present in the bicluster. Although its intuitiveness to show biclusters’ disposition, overlaps between bubbles do not necessarily correspond to real overlaps among biclusters. They are just an estimation of biclusters’ similarity. Bubble map visualization technique is usually used as a completion to help understanding the overall behavior of biclustering methods. Figure 11 shows an example of bubble map visualization technique.

Because heatmaps and parallel coordinates visualization techniques show their limits to draw high number of biclusters in the same view, especially with high rate of overlaps [31], some more sophisticated visualization techniques have been proposed based on a good combination between traditional gene expression visualization techniques, i.e., heatmaps and/or parallel coordinates, and sets visualization techniques [33], like *Venn-like diagrams* [31] and *node-link diagrams* [34].

### 4.4 Venn-like diagrams

*Euler* and *Venn diagrams* are considered as the oldest techniques to visualize sets and their intersections. They were introduced by John Venn in the 18th century and used as a common means of teaching set theory and logical relations in classrooms [35]. Based on an *area-proportional approach* where the drawn areas characterize the size of a set and its intersections, sets are represented by *closed curves* in the plane and set relations are illustrated by *curve overlaps*. All possible relations between sets, including intersection, inclusion, and exclusion, can be represented because there are no restrictions about the way to represent overlaps. *Venn diagrams* which are a particular form of Euler diagrams represent all possible intersections between sets, whether they are empty or not.

**Figure 12:**
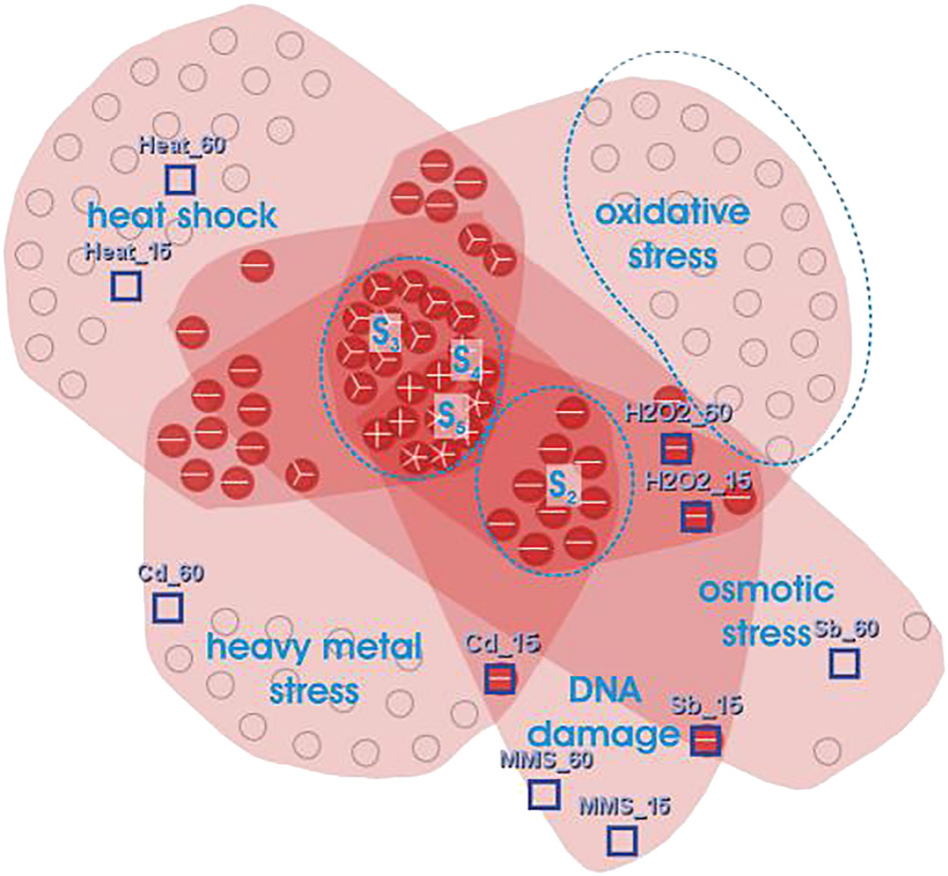
Visualization of five biclusters generated from stress gene expression data. Groups of biclusters and their overlaps can be deducted easily thanks to hulls representation [14].

Santamaría et al. [31] used a Venn-like representation where biclusters are represented as irregular surfaces, called *hulls*, and overlaps are represented by intersections of hulls. Groups of genes and conditions, either on just one bicluster or on specific overlaps, are represented by *glyphs*. A *glyph* is a pie chart divided into sectors whose numbers represent the number of biclusters containing genes and conditions. The size of a glyph represents the size of the corresponding group. The graph layout uses a force-directed algorithm where biclusters are represented by flexible overlapping groups of genes and conditions. Genes and conditions specific to a bicluster, or an overlap between biclusters, are represented by heatmaps and/or parallel coordinates in a separate view under demand. This method can deal with a good number of sparsely overlapping biclusters, but it suffers from low scalability especially by mid to high levels of biclustering overlaps. Figure 12 shows an illustration of this visualization technique.

### 4.5 Node-link diagrams

A *node-link diagram* is a 2D or 3D graph made-up of a set of nodes and edges. This type of data visualization represents entities as nodes or vertices and relationships between them as edges or links. In most cases, a node is represented by a circle and a link is represented by a line. To draw an understandable graph, the disposition of nodes and links needs to be well designed especially with high number of elements. Force-directed layout is usually used in such issues.

**Figure 13:**
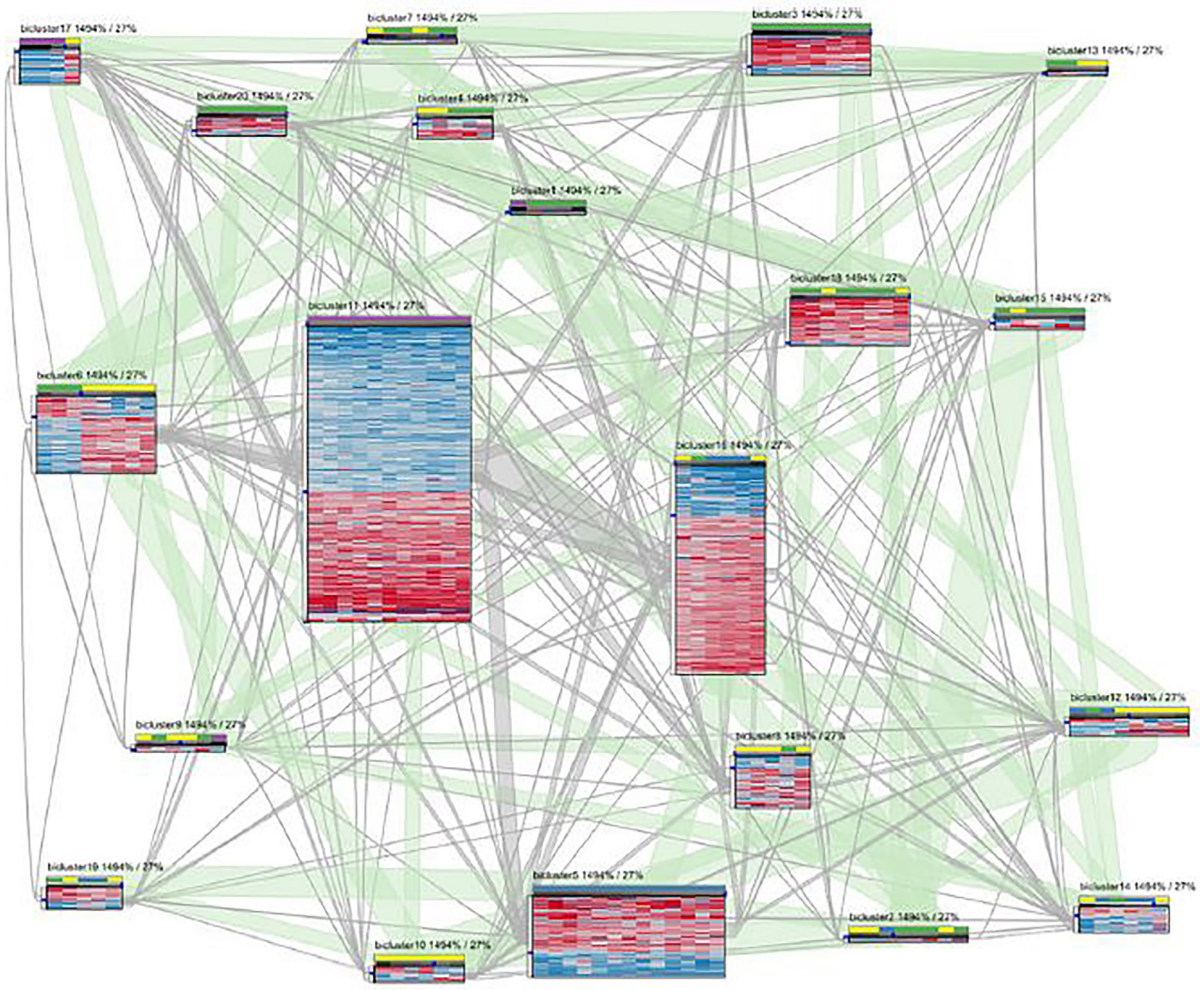
20 biclusters are visualized. Nodes represent the biclusters, drawn as heatmaps, and edges link the corresponding heatmaps at the position of the shared rows and columns [34].

Streit et al. [34] visualize biclusters, as well as their overlaps, by a *node-link graph*. Biclusters are the *nodes* of the graph while shared genes and conditions between biclusters are the *edges* or *bands*. Each bicluster node is represented by a heatmap matrix, where a row represents a gene and a column represents a condition of the corresponding bicluster. Overlaps between each pair of biclusters are encoded using edges that link the corresponding heatmaps at the position of the shared rows and columns. The thickness of an edge is proportional to the number of rows and columns shared by the linked biclusters. The graph layout uses a force-directed algorithm in which overlapping biclusters attract each other. Selecting a bicluster shows its details, such as its name or the labels of its corresponding genes and conditions. The proposed technique has a high rate of interactivity with a simple design based on heatmaps. Edges that encode overlaps give the user a clear vision with details about shared genes and conditions between each couple of biclusters. Because of the use of 1-on-1 visualization of overlaps, the multi-bicluster overlaps are difficult to identify with this method. Also, the scalability of this technique is low because, with a high level of overlaps, edges between biclusters will be too cluttered, rendering the full overview of the biclustering results impossible. Figure 13 illustrates an example of biclusters visualization based on this technique.

## 5 Evaluation of biclustering visualization techniques

We have made the evaluation of the surveyed techniques based on the three following principal criteria:

– *Dealing with overlaps*: minimize the number of superpositions of biclusters overlaps, by avoiding as much as possible that an overlap is drawn on another one.
– *Scalability*: maximize the number of biclusters drawn in the same view.
– *Clarity of visualization*: maximize the visibility of the displayed biclusters and their corresponding overlaps.

Based on the features of each surveyed technique, we can confirm that the geometrical limitations of both heatmaps and parallel coordinates [31] decrease remarkably the efficiency of these techniques, when referring to the mentioned evaluation criteria, especially the two first ones. Indeed, heatmaps are dimensionally unbalanced since generally the number of genes, around 10^3..44^ rows, is much higher than number of conditions, around 10^1…2^ columns [14]. So, replication techniques used to visualize several biclusters produce, in most cases, huge matrices. By the way, overlaps perception will be difficult and the scalability is limited. Also, most heatmaps matrices use a green-black-red color scale for the expression levels which is not distinguished by the human eye easily [7, 14]. Parallel coordinates technique is characterized by a high rate of cluttering caused by polylines’ overlaps when drawing some biclusters at once. Indeed, perception of overlaps is not straightforward and the scalability is not high. Visibility is good for perception of one bicluster since interpreting line patterns, such as parallel lines, mirror effects and changes in slope is straightforward for human eyes [14], but it is impossible when several large biclusters are visualized in the same parallel coordinates. The combination of heatmaps and/or parallel coordinates with more sophisticated set visualizations techniques, such as Venn diagrams [31] or node-link diagrams [34], improves considerably the scalability and the clarity of the visualization. Indeed, representing biclusters and their corresponding overlaps by hulls [31], or edges between heatmaps [34], will alleviate the representation by focusing on intersections between the visualized elements, i.e., biclusters, in a global overview and visualize details, i.e., gene expression levels, in a separate view as heatmaps or parallel coordinates. The scalability is also high with such techniques but with a lot of biclusters, and hence a lot of overlaps, the number of visualized biclusters in the same view will be in some cases impossible.

## 6 Conclusions

Compared to clustering, biclustering needs more sophisticated visualization techniques that should help in the analysis of gene expression data and, hence, lead to the extraction of nuggets of knowledge required by bioinformaticians. Visualization issues, such as scalability and overlaps between biclusters, are ones of the most important open directions for researchers. A sophisticated combination between traditional visualization techniques, like heatmaps or parallel coordinates, and one of the novel visualization techniques, mentioned in the literature [33], can be a solution to visualize biclustering results.
